# Targeted next-generation sequencing reveals recurrence-associated genomic alterations in early-stage non-small cell lung cancer

**DOI:** 10.18632/oncotarget.26349

**Published:** 2018-11-20

**Authors:** William C.S. Cho, Kien Thiam Tan, Victor W.S. Ma, Jacky Y.C. Li, Roger K.C. Ngan, Wah Cheuk, Timothy T.C. Yip, Yi-Ting Yang, Shu-Jen Chen

**Affiliations:** ^1^ Department of Clinical Oncology, Queen Elizabeth Hospital, Kowloon, Hong Kong; ^2^ ACT Genomics, Co. Ltd., Taipei, Taiwan; ^3^ Department of Clinical Oncology, The University of Hong Kong, Gleneagles Hong Kong Hospital, Wong Chuk Hang, Hong Kong; ^4^ Department of Pathology, Queen Elizabeth Hospital, Kowloon, Hong Kong; ^5^ ACT Genomics, Co. Ltd., Kowloon, Hong Kong

**Keywords:** biomarker, early-stage, lung cancer, next-generation sequencing, relapse

## Abstract

**Purpose:**

The identification of genomic alterations related to recurrence in early-stage non-small cell lung cancer (NSCLC) patients may help better stratify high-risk individuals and guide treatment strategies. This study aimed to identify the molecular biomarkers of recurrence in early-stage NSCLC.

**Results:**

Of the 42 tumors evaluable for genomic alterations, TP53 and EGFR were the most frequent alterations with population frequency 52.4% and 50.0%, respectively. Fusion genes were detected in four patients, which had lower mutational burden and relatively better genomic stability. EGFR mutation and fusion gene were mutually exclusive in this study. CDKN2A, FAS, SUFU and SMARCA4 genomic alterations were only observed in the relapsed patients. Increased copy number alteration index was observed in early relapsed patients. Among these genomic alterations, early-stage NSCLCs harboring CDKN2A, FAS, SUFU and SMARCA4 genomic alterations were found to be significantly associated with recurrence. Some of these new findings were validated using The Cancer Genome Atlas (TCGA) dataset.

**Conclusions:**

The genomic alterations of CDKN2A, FAS, SUFU and SMARCA4 in early-stage NSCLC are found to be associated with recurrence, but confirmation in a larger independent cohort is required to define the clinical impact.

**Materials and Methods:**

Paired primary tumor and normal lung tissue samples were collected for targeted next-generation sequencing analysis. A panel targets exons for 440 genes was used to assess the mutational and copy number status of selected genes in three clinically relevant groups of stage I/II NSCLC patients: 1) Early relapse; 2) Late relapse; and 3) No relapse.

## INTRODUCTION

Lung cancer is the leading cause of cancer-related mortality in the world, and non-small cell lung cancer (NSCLC) accounts for 80–85% of all lung cancers [1]. Current treatment for NSCLC is mainly based on the clinical staging systems in addition to some factors, such as smoking history, gender and genetic mutation [2, 3]. The 5-year survival rate of NSCLC patients is about 30–60%, with local recurrence and metastasis being the leading causes of death [4]. Stage I/II NSCLC patients are typically treated with complete surgical resection of the tumor, over half of the patients with early-stage NSCLC will never recur after surgical resections. However, individual clinical outcome varies widely. Even after the entire resection of tumor, 30–55% of patients will develop disease recurrence within the first five years of surgery and ultimately die of the disease [4–8]. Unfortunately, the molecular mechanisms underlying recurrence remain unclear. Identification of patients with high risk of recurrence may help to guide more tailored treatment plan for the individual patient, in particular for those who may receive additional benefit from adjuvant therapy and targeted therapy.

In recent years, high-throughput genomic analysis has been used to obtain genomic information of NSCLC. With the advancement of next-generation sequencing (NGS), it is now possible to identify the oncogenic alterations that will previously be missed by conventional pathological diagnosis. Rather than sequencing the entire genome or exome, a clinical cancer gene test (which includes a panel of genes that show frequent mutations in cancer) can reduce the amount of specimen, time and effort required to perform sequencing. These tests usually use a polymerase chain reaction capture-based or amplicon-based NGS assay for the deep targeted sequencing of the genes of interest in limited biological specimens [9–11]. Our study performed this kind of targeted NGS to identify the genomic alterations associated with recurrence in early-stage NSCLC patients. The identification of these genomic alterations may help to distinguish patients at higher risk and guide treatment strategies for early-stage NSCLC.

## RESULTS

We identified 45 pathologically confirmed stage I/II NSCLC patients with available formalin-fixed paraffin-embedded (FFPE) tissues. Of these, three tumor specimens were excluded due to the insufficiency of tumor cells or tumor purity was lower than acceptance criteria. 42 patients were included in NGS analysis. At the time of analysis, 16 patients were reported having early relapse (ER), 15 patients having late relapse (LR) and 11 patients having no relapse (NR). The general characteristics of the study population are summarized in Table 1. The median follow-up time of NR patients from the date of surgery until the last follow-up or the end of the study was 50.4 months.

**Table 1:** Patient characteristics

Characteristics	Number	%
Patients (*n*)	42	
Sex		
Male	26	61.9
Female	16	38.1
Age, median (range, year)	65	(46–85)
Stage		
T1	38	90.5
T2	4	9.5
Smoking status		
Non-smoker	19	45.2
Ex-smoker	17	40.5
Current smoker	5	11.9
Not mention	1	2.4
Recurrence		
Early relapse	16	38.1
Late relapse	15	35.7
No relapse	11	26.2

### Genomic alterations

**Figure 1:**
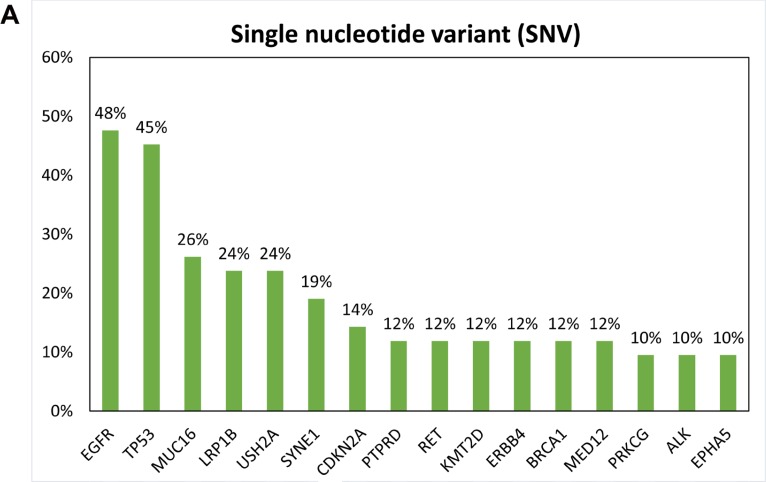

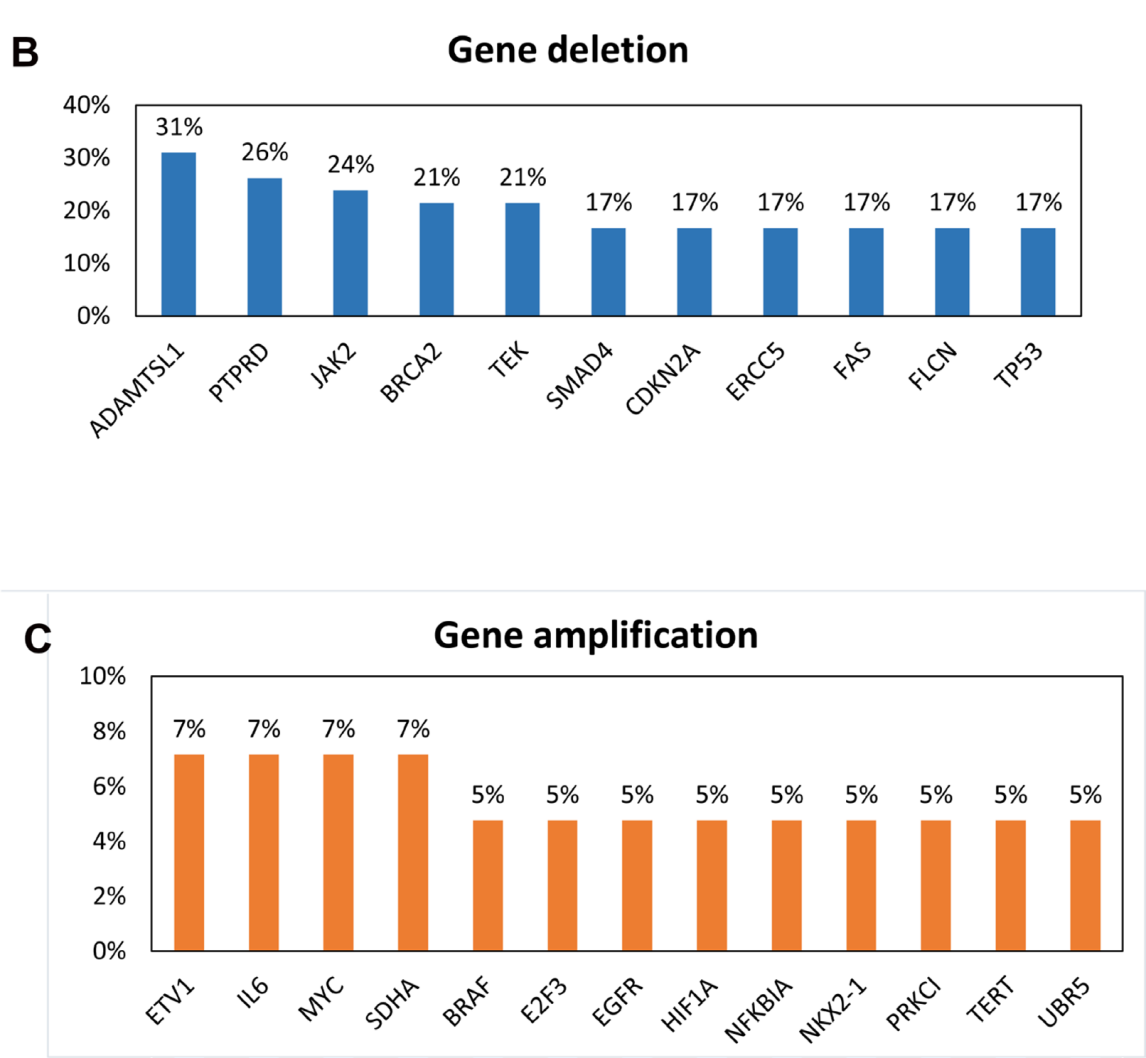
Frequency of genomic alterations in each gene Top genes of **(A)** single nucleotide variant, copy number alteration of **(B)** deletion and **(C)** amplification are sorted by the total frequency of alteration events on the x-axis.

**Figure 2:**
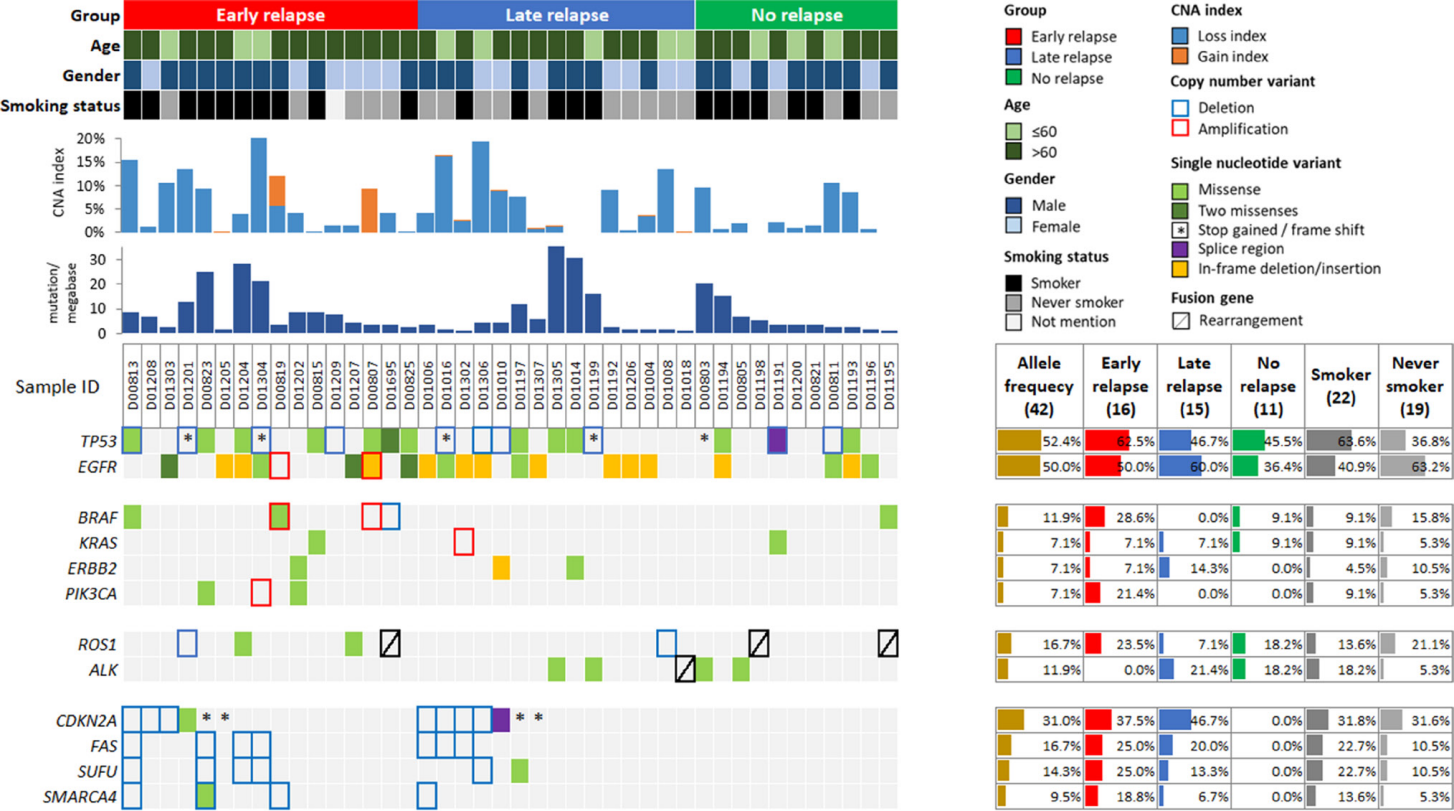
Genomic alterations identified in early-stage non-small cell lung cancer Tumor samples of early relapse, late relapse, and no relapse are arranged from left to right. Patient information is displayed on the top panel, including group, age, gender, and smoking status. Tumor mutational number and copy number alteration (CNA) index are shown below the patient information. Genomic alterations are annotated according to the color panel on the top right corner of the image. Mutation rates of each gene are plotted on the right of the genomic alterations.

Genomic alterations were identified in 324 (73.6%) of 440 targeted genes, including 180 (40.9%) of single nucleotide variants (SNVs), 193 (43.9%) of gene deletions and 92 (20.9%) of gene amplifications (Supplementary Table 1). The genomic alterations with the highest mutated frequencies were shown in Figure 1. Gene deletion was more common event than gene amplification in this study (Figure 2, copy number variant (CNV) index). *TP53* and *EGFR* were the most frequent alterations detected in this study, with a genomic alteration frequency of 52.4% and 50%, respectively (Figure 2). *EGFR* exon 19 in-frame deletion was the most common *EGFR* mutation (11 cases), followed by activating mutation L858R in exon 21 (5 cases), wild-type *EGFR* amplification (2 cases), and exon 20 insertion (1 case). Other *EGFR* mutations were also detected, including K860I, A871G, L861Q, G719S, V592I and T302H. Interestingly, two coexisting *EGFR* alterations were found in 4 ER patients (D00807, D00825, D01207 and D01303). The L858R mutation occurred in *cis* with A871G and K860I, whereas the allelic relationship of G791S and L861Q mutations were unknown (D01303) due to the limited read length of NGS platform. No T790M point mutation was observed (Table 2).

**Table 2:** *EGFR* alterations in the patients

Sample ID	Genomic alteration	Gender	Age	Smoking status	Stage	Relapse
**D00807**	Amplification, 767_V769dup	F	61	Non-smoker	IA (T_1_N_0_)	Early
**D00819**	Amplification	M	69	Ex-smoker	IB (T_2a_N_0_)	Early
**D00825**	L858R, K860I	M	79	Ex-smoker	IB (T_2a_N_0_)	Early
**D01204**	E746_A750del	M	43	Ex-smoker	IB (T_2a_N_0_)	Early
**D01205**	L747_A750delinsP	M	62	Current smoker	IA (pT_1a_N_0_)	Early
**D01207**	L858R, A871G	F	64	Non-smoker	IIA (pT_2a_N_1_)	Early
**D01303**	G719S, L861Q	M	47	Non-smoker	IB (pT_2a_N_0_)	Early
**D01304**	V592I	M	57	Ex-smoker	IB (T_2a_N_0_)	Early
**D01004**	E746_A750del	M	77	Non-smoker	IB (T_2a_N_0_)	Late
**D01006**	E746_A750del	M	74	Non-smoker	IIB (T_2_N_1_)	Late
**D01016**	L858R	M	52	Non-smoker	IIB (T_2_N_1_)	Late
**D01192**	E746_A750del	F	70	Non-smoker	IA (T_1_N_0_)	Late
**D01197**	T302H	M	63	Ex-smoker	IA (T_1b_N_0_)	Late
**D01206**	E746_A750del	F	62	Non-smoker	IA (pT_1b_N_0_)	Late
**D01302**	E746_A750del	M	65	Ex-smoker	IA (pT_1b_N_0_)	Late
**D01306**	L747_P753delinsS	F	61	Non-smoker	IB (pT_2_N_0_)	Late
**D01307**	E746_A750del	F	71	Non-smoker	IA (pT_1b_N_0_)	Late
**D00811**	L858R	M	58	Non-smoker	IB (T_2a_N_0_)	No
**D01193**	E746_A750del	M	81	Ex-smoker	IA (pT_1b_)	No
**D01194**	L747_T751del	M	65	Current smoker	IA (T_1_N_0_)	No
**D01196**	L858R	F	76	Non-smoker	IA (T_1b_N_0_)	No

*KRAS* G12C, amplification and G12V were detected in 3 relapsed patients. *ERBB2* V659E, E507K and exon 20 insertion (A775_G776insYVMA) were also found in 3 relapsed patients. *ERBB2* mutations were mutated exclusively from *EGFR*. *PIK3CA* genomic alterations were only found in ER patients, including mutation type I459V and H1047R, and also amplification. *BRAF* mutation was identified in 4 cases, including mutation type K601E, G219A and G469A, as well as amplification and deletion.

Fusion genes (*SDC4-ROS1*, *EML4-ALK*, *SDC4-ROS1*, *EZR-ROS1*) were detected in 4 patients, whose tumors had lower mutation burden and demonstrated relatively better genome stability with extremely low copy number alteration (CNA) index (Figure 2). *EGFR* mutation and fusion gene were mutually exclusive in this study.

### Genomic alterations absent in the NR patients

*CDKN2A, FAS* and *SUFU* were the most frequently altered genes detected in the relapsed patients, with a population frequency of 31.0% (13 cases), 16.7% (7 cases) and 14.3% (6 cases), respectively. A total of five loss-of-function (LOF) mutations (E61*, R80*, R58*, E61 and P114L) and one variant of unknown significance (VUS) in the splice region of *CDKN2A* were detected in the relapsed patients, whereas the deletions of *CDKN2A* were found in seven relapsed patients. A *FAS* deletion was detected in 7 relapsed (16.7%) cases, and no nucleotide variant was detected. *SUFU* genomic alterations were found in 6 relapsed patients (14.2%), including 5 deletions and a VUS missense mutation (D159E). *SMARCA4* variants were detected in 4 relapsed (9.5%) cases, including a VUS (G1159L) and deletions (Figure 2).

### Tumor mutational burden (TMB) and CNA index

**Figure 3:**
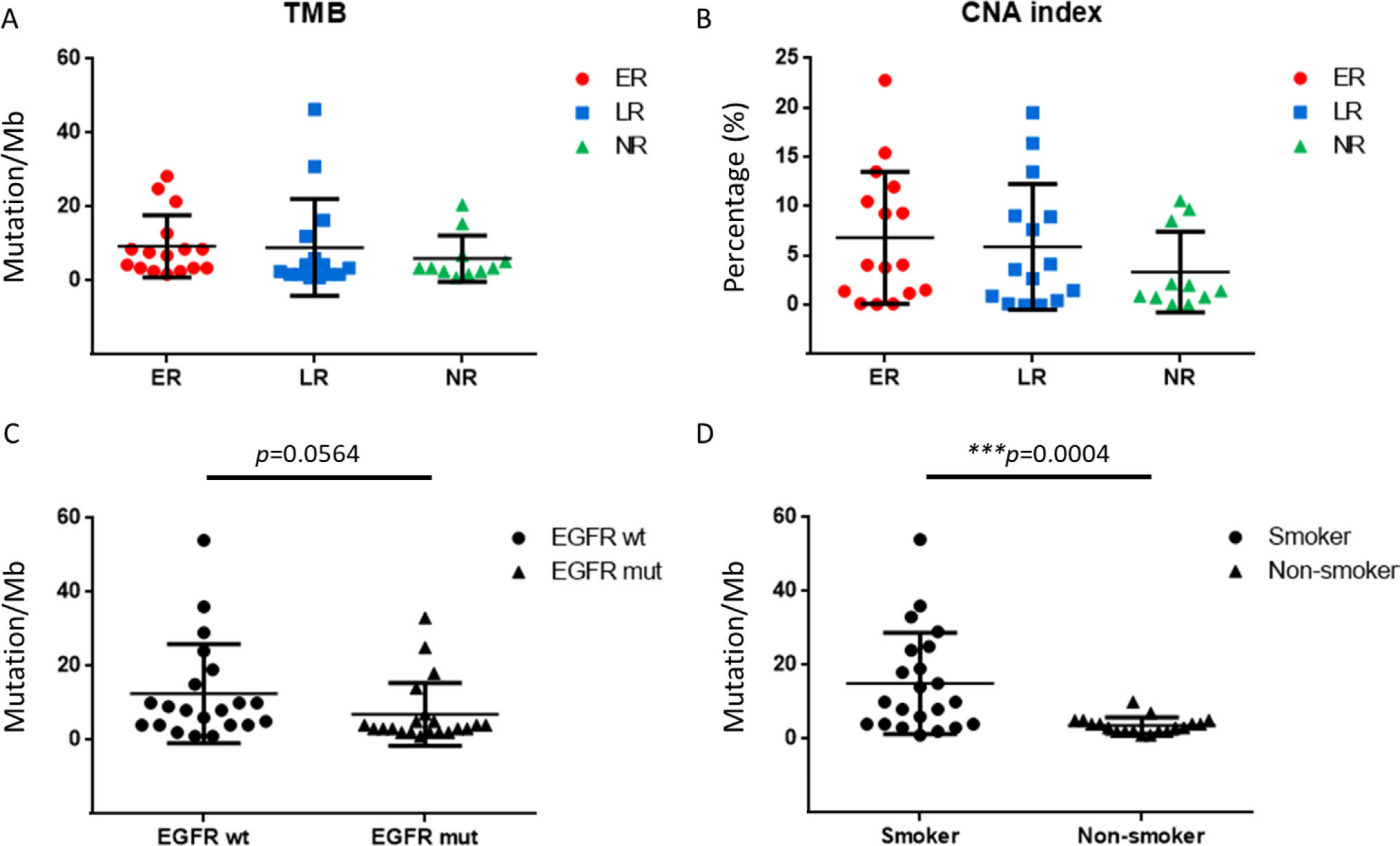
Tumor mutational burden (TMB) was highly correlated with smoking status **(A)** TMB did not show significant differences among early relapse (ER), late relapse (LR), and no relapse (NR) patients. **(B)** Copy number alteration (CNA) index was not correlated with relapse status, whereas CNA index was increased in ER patients. **(C)** TMB is of borderline significance with *EGFR* mutations (*p* = 0.0564). **(D)** TMB was highly associated with smoking status (^***^p = 0.0004).

**Figure 4:**
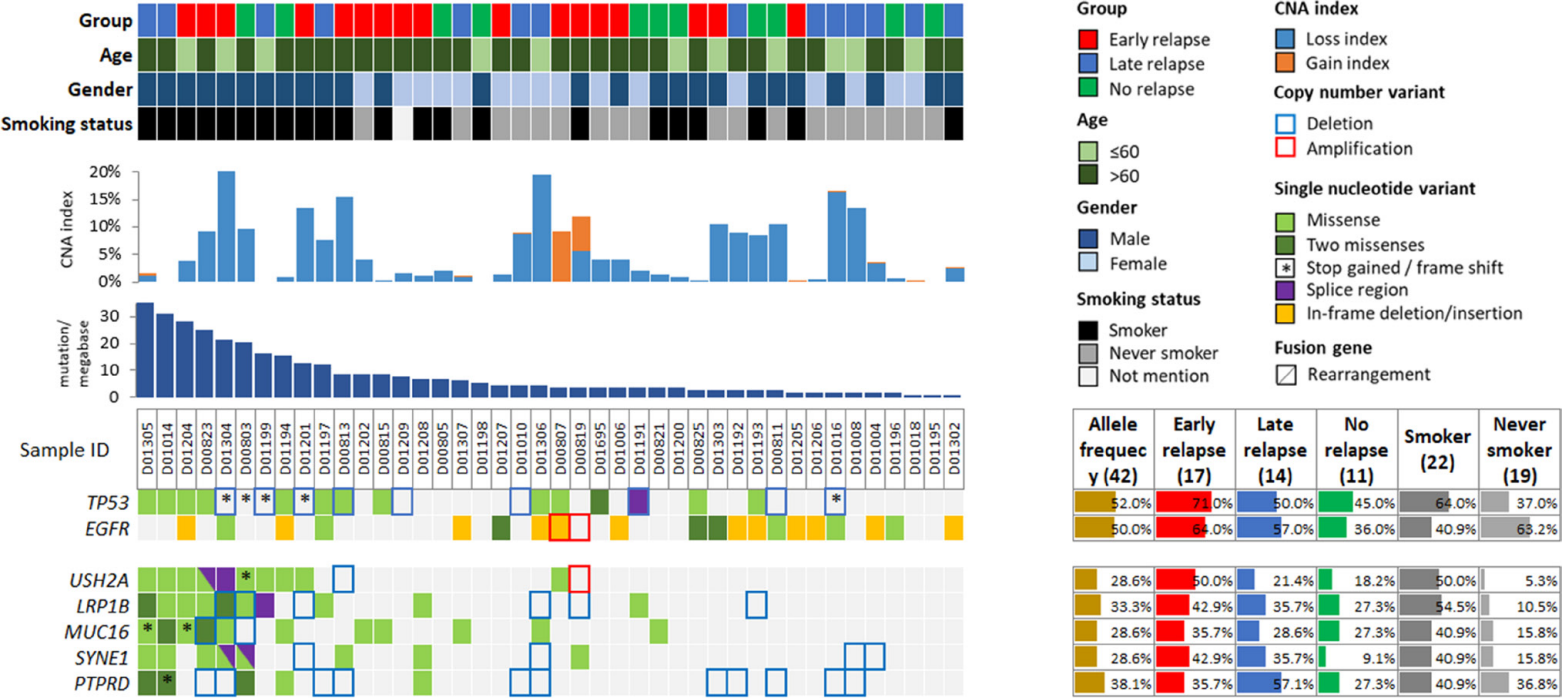
Genomic alterations identified in high tumor mutational burden (TMB) patients Tumor samples of high TMB are arranged from left to right. Patient information is displayed on the top panel, including group, age, gender, and smoking status. TMB and copy number alteration (CNA) index are shown below the patient information. Genomic alterations are annotated according to the color panel on the top right corner of the image. Alteration rates of each gene are plotted on the right of the genomic alterations.

The median TMB was 3.86 mutations per megabase (Mb), with a range of 0.86∼46.31 mutations/Mb in this subset of patients (Table 3). The average values of TMB were 9.33, 8.98 and 6.00 mutations/Mb in ER, LR, and NR patients, respectively. The median of TMB were 7.29, 3.43 and 3.43 mutations/Mb in ER, LR and NR patients, respectively. The range of CNA index was between 0∼22.79% and the average index values were 6.81%, 5.89% and 3.34% in the ER, LR and NR patients, respectively. The TMB and CNA index were not significantly associated with relapse status, while both TMB and CNA indices were increased in the ER patients (Figure 3A and 3B). Furthermore, TMB was highly associated with smoking status (^***^p = 0.0004), and borderline significance was associated with *EGFR* alterations (*p* = 0.0564) (Figure 3C and 3D). Four *EGFR* mutated patients with high TMB were all smokers. Most of the high TMB patients were male smokers in this study. Several genes were found to cluster with smoking status and TMB, including *TP53*, *USH2A*, *LRP1B*, *MUC16* and *SYNE1* (Figure 4).

**Table 3:** Distribution of tumor mutational burden (TMB)

Mutation/Megabase	All patients	%	Early relapse	%	Late relapse	%	No relapse	%
0.86	3	7.1	0	0	2	4.8	1	2.4
1.72	6	14.3	1	2.4	4	9.5	1	2.4
2.57	5	11.9	2	4.8	1	2.4	2	4.8
3.43	7	16.7	3	7.1	1	2.4	3	7.1
4.29	3	7.1	1	2.4	2	4.8	0	0
5.15	1	2.4	0	0	0	0	1	2.4
6.00	1	2.4	0	0	1	2.4	0	0
6.86	2	4.8	1	2.4	0	0	1	2.4
7.72	1	2.4	1	2.4	0	0	0	0
8.58	3	7.1	3	7.1	0	0	0	0
12.01	1	2.4	0	0	1	2.4	0	0
12.86	1	2.4	1	2.4	0	0	0	0
15.44	1	2.4	0	0	0	0	1	2.4
16.30	1	2.4	0	0	1	2.4	0	0
20.58	1	2.4	0	0	0	0	1	2.4
21.44	1	2.4	1	2.4	0	0	0	0
24.87	1	2.4	1	2.4	0	0	0	0
28.30	1	2.4	1	2.4	0	0	0	0
30.87	1	2.4	0	0	1	2.4	0	0
46.31	1	2.4	0	0	1	2.4	0	0
Total patients	42	100	16	38.1	15	35.7	11	26.2

### Genomic alterations associated with the risk of recurrence

**Figure 5:**
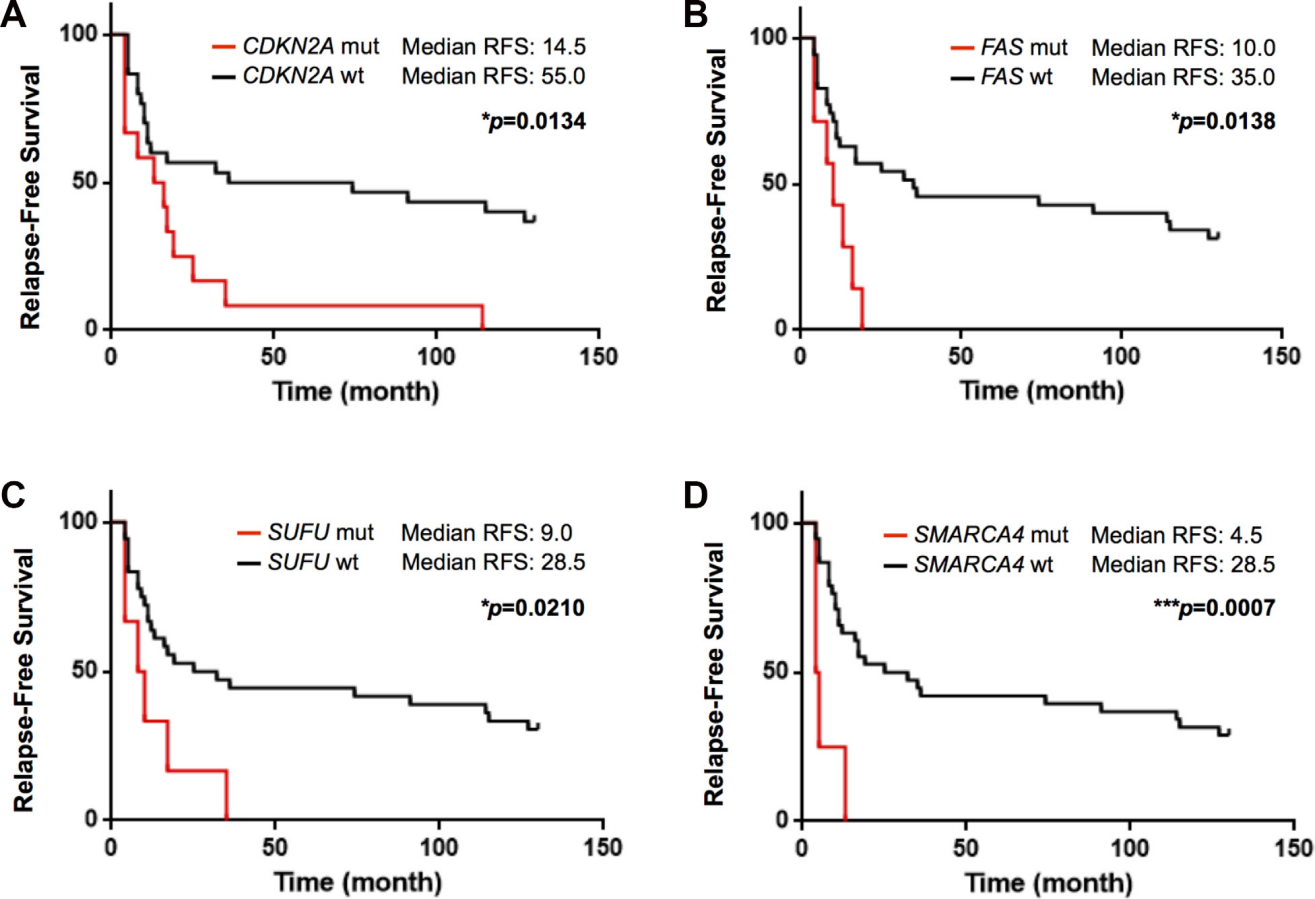
Kaplan–Meier curve of relapse-free survival according to mutational status in this study The alterations of **(A)** *CDKN2A*, **(B)** *FAS,* **(C)** *SUFU* and **(D)** *SMARCA4* showed shorter median relapse-free survival and all the alterations were significantly associated with an increased risk of recurrence in early-stage NSCLC (Gehan-Breslow-Wilcoxon test). ^*^p < 0.05, ^***^p < 0.001.

Further analysis found that the mutations of *CDKN2A*, *FAS*, *SUFU* and *SMARCA4* were significantly associated with an increased risk of recurrence in early-stage NSCLC (Figure 5). The median time to recurrence was 14.5 months in the *CDKN2A*-mutated patients and 55.0 months in the *CDKN2A* normal patients (*p* = 0.0134). The median time to recurrence was 10.0 months in the *FAS* loss patients and 35.0 months in the *FAS* normal patients (*p* = 0.0138). The median time to recurrence was 9.0 months in the *SUFU*-mutated patients and 28.5 months in the *SUFU* normal patients (*p* = 0.0210). The median time to recurrence was 4.5 months in the *SMARCA4*-mutated patients and 28.5 months in the *SMARCA4* normal patients (*p* = 0.0007). In order to evaluate if the genomic alterations identified in *CDKN2A*, *FAS*, *SUFU* and *SMARCA4* genes are significantly associated with early relapse, multivariate Cox regression model was tested. Our results revealed that the genomic alterations of *CDKN2A*, *FAS*, *SUFU* and *SMARCA4* are independent risk factors significantly associated with the risk of recurrence regardless of age, gender, stage and smoking status (Table 4).

**Table 4:** Univariate and multivariate analysis of the risk factors associated with disease recurrence

Mutation	Total (%)	Median time to recurrence (months)	Univariate test	Multivariate test			
				This cohort		TCGA cohort	
			*p* value^a^	HR^b^ (95% CI)	*p* value^c^	HR^b^ (95% CI)	*p* value^c^
*CDKN2A*							
Wild-type	29 (69.0)	55.0	0.0134	1	0.011	1	0.931
Mutant	13 (31.0)	14.5		2.927 (1,278–6.702)		0.985 (0.701–1.384)	
*FAS*							
Wild-type	35 (83.3)	35.0	0.0138	1	0.010	1	0.033
Mutant	7 (16.7)	10.0		4.835 (1.462–15.992)		1.461 (1.031–2.070)	
*SUFU*							
Wild-type	38 (90.5)	28.5	0.0210	1	0.010	1	0.017
Mutant	4 (9.5)	9.0		3.505 (1.342–9.154)		1.528 (1.080–2.163)	
*SMARCA4*							
Wild-type	38 (90.5)	28.5	0.0007	1	0.009	1	0.681
Mutant	4 (9.5)	4.5		6.844 (1.630–28.726)		1.074 (0.764–1.509)	

^a^ Gehan-Breslow-Wilcoxon test.

^b^ Cox proportional hazards regression model based on age, gender, stage and smoking status.

^c^ *P* value for multivariate Cox proportional hazard regression.

### Validation of findings in The Cancer Genome Atlas (TCGA) dataset

**Figure 6:**
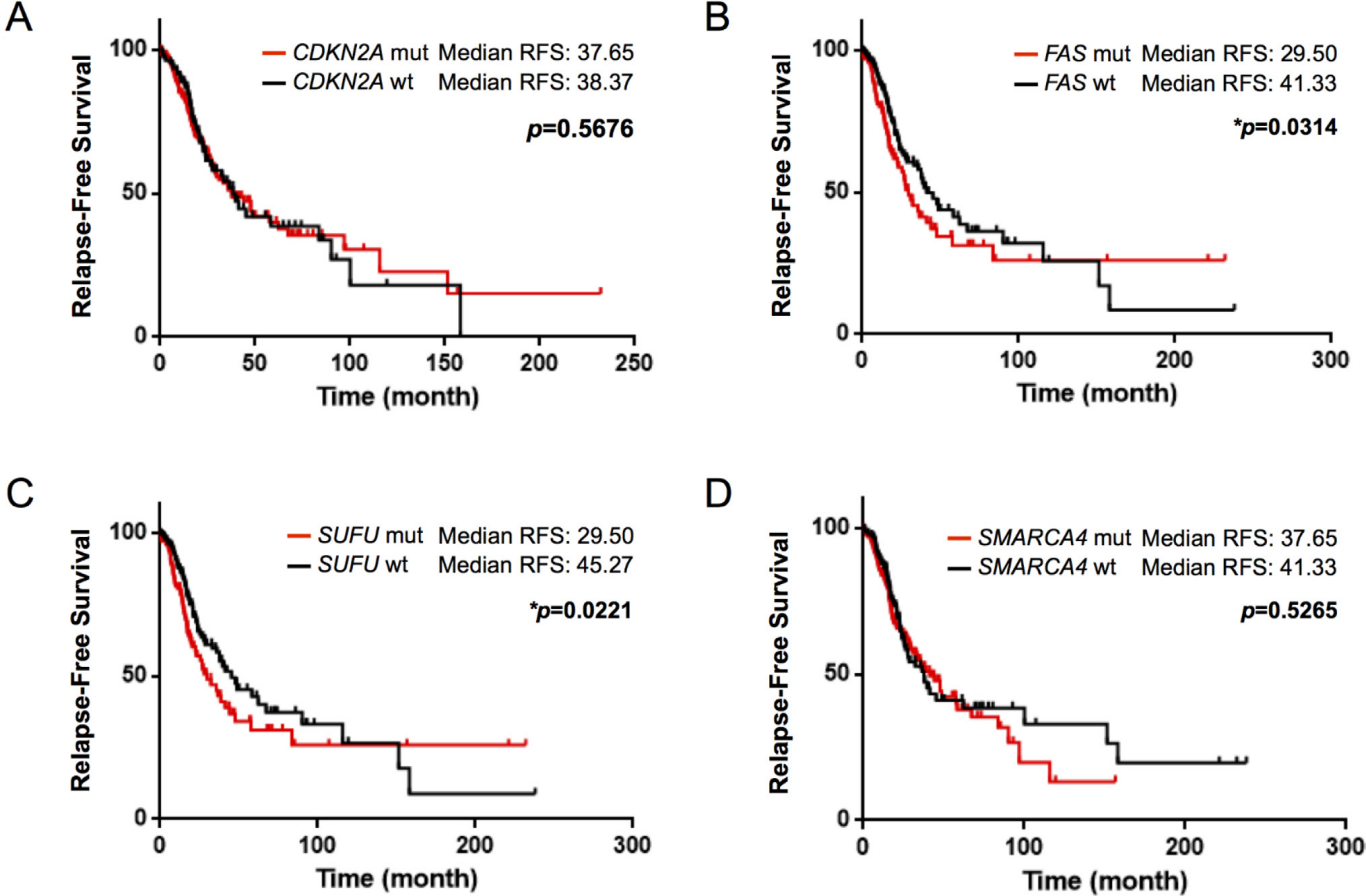
Kaplan–Meier curve of relapse-free survival according to mutational status in the TCGA cohort (TCGA, Provisional) The alterations of (**A**) *CDKN2A*, (**B**) *FAS*, (**C**) *SUFU* and (**D**) *SMARCA4* showed shorter median relapse-free survival. Only *FAS* and *SUFU* genes were significantly associated with an increased risk of recurrence in early-stage NSCLC (Gehan-Breslow-Wilcoxon test). ^*^p < 0.05.

We aimed to validate our findings in a publically available TCGA dataset. In the TCGA dataset (TCGA, Provisional), 177 out of 283 (62.5%) stage I/II NSCLC patients showed *CDKN2A* mutation, and the median relapse-free survival (RFS) was 37.65 months in the *CDKN2A*-altered patients and 38.37 months in the *CDKN2A* normal patients (*p* = 0.5676). We also analyzed the TCGA dataset for the presence of *FAS*, *SUFU* and *SMARCA4* alterations in recurrent NSCLC. A total of 95 out of 268 (35.4%) showed a deletion of *FAS*, 97 out of 269 (36.1%) showed an alteration of *SUFU*, and 161 out of 300 (53.7%) showed a mutation of *SMARCA4*, with Gehan-Breslow-Wilcoxon test *p* value of 0.0314, 0.0221 and 0.5265, respectively. RFS was shorter in patients with genomic alterations in *CDKN2A*, *FAS, SUFU* and *SMARCA4* genes (Figure 6). Using Cox regression model for multivariate analysis, we found that only *FAS* and *SUFU* mutants were significantly associated with the risk of recurrence (Table 4).

## DISCUSSION

Local recurrence or metastasis is generally believed to account for the failure of therapy and recurrence after initial surgical resection. Although early-stage NSCLC patients have better prognosis, nearly 30–35% of them will relapse [12–14]. Owing to the fact that NSCLC has heterogeneous histopathological features, conventional classification systems (such as tumor-nodes-metastasis staging) cannot fully explain its clinical behaviors [15]. Regarding the unsatisfactory survival rates, many researchers try to look for possible methods that can help to predict the outcome of early-stage patients. The tumor size of the invasive component has been reported to be an important prognostic factor for early-stage adenocarcinoma [16, 17]. In addition to low-dose computed tomography, many biomarkers (especially molecular biomarkers) have been developed to supplement clinical diagnosis, such as carcinoembryonic antigen level [18–20], epigenetic modifications [21, 22], gene-expression profiles [23–25] and the detection of genomic alterations [26–29]. However, few studies have explored the genomic alterations of early-stage NSCLC patients [20, 26, 30–32]. Contemporary lung cancer research has distincted itself from traditional study with an unprecedented large amount of data and tremendous diagnostic and therapeutic innovations. Data are currently generated in high-throughput fashions with the integration and application of sequencing analysis. The application of NGS on mutational analysis gives us a more comprehensive genomic landscape in NSCLC. If we can identify early-stage NSCLC patient with a high risk of recurrence, we are able to select a more appropriate candidate treatment (such as adjuvant therapy or targeted therapy). The ability to identify patients with high-risk of early recurrence following surgical resection can also help formulating more intensive surveillance schedule, performing more extensive surgery including lymph node dissection, instituting adjuvant postoperative chemotherapy and other adjuvant personalized or targeted therapy for these patients [12, 33].

In this study, we aimed to investigate the impact of genomic alterations on the recurrence of early-stage NSCLC. Oncogenic mutations were detected more frequently in the ER patients than in the LR and NR patients. Two coexisting *EGFR* mutations representing two oncogenic drivers were found in some ER patients, but not in the LR and NR patients. Some genomic alterations were absent in the NR patients, whereas some mutations were found only in the ER patients. These findings add to the growing evidence that genomic alterations may be a significant factor for recurrence in early-stage NSCLC. Several rare *EGFR* mutations were detected in the extracellular domain (V592I and T302H) and tyrosine kinase domain (A871G, K860I, G719S, and L861Q), suggesting NGS is a promising and comprehensive platform to detect multiple alterations simultaneously. Our results have provided a genome-wide view on the genomic landscape of early-stage NSCLC, including TMB and CNA burden, which allows us to correlate genomic profiles to the risk of recurrence. Consistent with a previous study [34], we observed genomic alterations in several known prognostic biomarkers. In addition, we identified some uncommon but potentially actionable mutations in early-stage NSCLC.

This study is unique in that it identified 4 (*CDKN2A*, *FAS*, *SUFU*, and *SMARCA4*) potential genomic biomarkers of recurrence in early-stage NSCLC. We found *CDKN2A* to be the most frequently altered genes detected in the relapsed patients. Indeed, *CDKN2A* status was found to be associated with various cancers [35–40]. A recent study identified *CDKN2A* as a tumor suppressor whose inactivation promoted homotypic cell-in-cell formation in human cancer cells [41]. Our analysis also suggested that *CDKN2A* might be a potential biomarker of recurrence in early-stage NSCLC.

*FAS* gene has been reported to be associated with tumor progression. The previous study has provided evidence that Fas was important for natural killer cell-mediated immune surveillance and chemosensitivity. Their model for Fas LOF in tumor progression showed that Fas and FasL interactions were important in the control of malignant disease and that changes in the level of Fas expression could determine immune escape and therapeutic responses [42]. We have identified *FAS* as a potential biomarker of recurrence, as our results showed that *FAS* mutation was significantly associated with recurrence in early-stage NSCLC.

*SUFU* (suppressor of fused) is an important negative regulator of the Hedgehog (HH) pathway [43]. Activation of HH pathway signaling has been reported in various cancers, including cancers of the lung, skin, colon and stomach, which is involved in cancer cell proliferation and metastasis [44]. Moreover, several studies have demonstrated that the HH pathway is activated in NSCLC [45–48]. However, the prognostic roles of *SUFU* in lung cancer have not been addressed. Further study to clarify whether *SUFU* is indeed involved in HH pathway activation or ER in lung cancer would be interesting.

Alteration in *SMARCA4* was detected only in the relapsed patients. Low expression of *SMARCA4* has been reported to be associated with worse prognosis and is supposed to be a predictive biomarker for increased sensitivity to platinum-based chemotherapy in NSCLC [49]. In this study, we found that the loss of *SMARCA4* was only detected in the relapsed patients and thus might serve as a potential biomarker for recurrence in early-stage NSCLC.

On the other hand, *USH2A*, *LRP1B*, *MUC16*, and *SYNE1* genes have been reported to have frequently mutated in various cancer types, particularly lung adenocarcinoma and lung squamous cell carcinoma [50, 51]. However, the functional roles of these genes in tumorigenesis remain unclear. We noted that the length of proteins coded by *USH2A*, *LRP1B*, *MUC16* and *SYNE1* were of 5202, 4599, 14507 and 8797 amino acids, respectively. A recent investigation has addressed that mutation rate was higher in the genes coding for proteins of longer length. For example, extremely long genes such as TTN have a high mutational frequency (52%) in lung squamous TCGA data [52]. Therefore, further study is required to clarify the biological meaning of these alterations.

There are a number of advantages of using targeted NGS panel for sequencing analysis [53]. It can reduce the amount of specimen, time, and effort required to perform deep targeted sequencing. However, it also had some limitations. Targeted NGS may have missed smaller region copy number alterations and other mutations in regions not covered by the panel. We tried to avoid this by using a panel consisted of as many as 440 genes that showed frequent mutations in cancer and thus could detect the genomic alterations of interest. The detection of genomic alterations in some known prognostic biomarkers confirmed the reliability of our panel.

In conclusion, our new findings revealed that genomic mutations, single nucleotide variant and CNV might play a role in the clinical outcomes of early-stage NSCLC patients. In addition, our results also indicated that the mutated genes might serve as potential biomarkers of recurrence. Notably, *CDKN2A*, *FAS*, *SUFU* and *SMARCA4* mutations were significantly associated with an increased risk of recurrence in early-stage NSCLC. Our results suggest that utilizing these genomic alterations to guide additional adjuvant therapies after surgery may improve outcomes in selected patients with high-risk of recurrent disease. Although some of our findings have been validated using TCGA dataset, confirmation in a larger independent cohort of the Asian population is warranted.

## MATERIALS AND METHODS

### Patient selection

We used a cohort of patients with pathologically confirmed stage I/II (AJCC 7th edition) NSCLC who underwent lobectomy and/or thoracotomy at Queen Elizabeth Hospital (Hong Kong) between March 2004 and March 2015 (Supplementary Table 2). Patients with early-stage NSCLC who developed recurrence ≤ 1 year after treatment will be included in this study. In addition, the tissues of patients with early-stage NSCLC who did not develop recurrence ≤ 1 year after treatment will be included as a control. We defined ER as patients who relapsed within one year of treatment. LR were those patients who did not develop recurrence within one year of treatment. NR were those patients without evidence of recurrence after treatment on the date of last follow-up or before the end of the study.

### Sample collection

The archival FFPE tissue samples of patients with pathologically confirmed stage I/II NSCLC were collected for NGS analysis. Paired primary tumor and normal lung tissue samples were included to identify somatic mutations. This study was approved by the Kowloon Central/Kowloon East Cluster Research Ethics Committee (Hospital Authority, Hong Kong).

### Sample preparation

One 5 μm hematoxylin and eosin stained slide and ten 10 μm unstained slides were prepared. Samples needed to be > 25 mm^2^ and tumor cell content > 30% tumor cells were considered eligible for targeted NGS analysis.

### DNA and RNA extraction

Ten 10 μm lung tissue sections were obtained from each patient. Genomic DNA and RNA were isolated from FFPE tissue sections with the RecoverAll^™^ Total Nucleic Acid Isolation Kit (Invitrogen, MA, USA) according to manufacturer’s protocol. Extracted DNA and RNA concentration were measured by Qubit-iT^™^ dsDNA HS Assay Kit (Invitrogen) and Qubit RNA HS Assay Kit (Invitrogen), respectively. The integrity of DNA and RNA were assessed using the Fragment Analyzer (Advanced Analytical Technologies, Inc, IA, USA).

### Next-generation sequencing of genomic DNA

Targeted deep NGS was used to assess the mutational status, single nucleotide variant, small insertions and deletions and copy number variant of 440 cancer-related genes (Supplementary Table 3) (ACT Genomics, Taiwan). Extracted genomic DNA from FFPE was amplified using 18,700 primer pairs targeting selected genes. Amplicons were constructed with barcoded libraries using the Ion AmpliSeq Library Kit (Life Technologies, MA, USA). Sequencing was performed on the Ion Proton sequencer (Life Technologies) according to the manufacturer’s protocol. The mean sequencing depth was more than 700x, and the mean uniformity was more than 75%.

### NGS data analysis

For the 440-gene panel, sequencing raw reads were mapped to the hg19 human reference genome using Torrent Suite Server version 5.2, base calling and variant calling were performed with the Torrent Suite Variant Caller plug-in version 5.2. The Ion Torrent default pipeline and parameters were used for data analysis. Variants reported in 1000 Genomes Project Phase 3 with > 1% minor allele frequency (Asian populations) were considered as polymorphisms and excluded from further analysis. Variants detected in 25 peripheral blood mononuclear cell (PBMC) in-house samples from healthy volunteers were also disregarded as SNPs or technical errors. Variants with coverage ≥ 25, allele frequency ≥ 5% were retained. CNV was analyzed using ONCOCNV (https://github.com/BoevaLab/ONCOCNV) [54]. The diploid reference baseline was established according to our in-house PBMC samples from healthy volunteers. ADTEx was applied for estimating tumor purity and correcting baseline shifts based on SNP information [55]. Copy number amplification was defined as an observed copy number ≥ 10, whereas copy number loss was defined as an observed copy number ≤ 1. Paired primary tumor and normal lung tissue samples were compared to identify tumor somatic mutations. TMB was calculated according to the number of detected mutations and the number of analyzed base pairs (1.166 Mb). CNA index was calculated by the percentage of the regions of genes altered in a tumor and the total regions of the genes that covered in the test on the chromosome to measure degree of genomic instability across the entire genome of a tumor. Statistical analysis of TMB and CNA index distribution with differences between each group was assessed as unpaired *t-*test using GraphPad Prism (v.6.0; GraphPad Inc., CA, USA).

### Fusion gene test

Fusion transcripts for *ALK*, *ROS1*, *RET* and *NTRK* genes were tested for genetic rearrangement. Extracted RNA was reverse transcribed using SuperScript VILO cDNA Synthesis Kit (Invitrogen) according to the manufacturer’s instructions. The library was constructed using the Ion AmpliSeq^™^ RNA Fusion Lung Cancer Research Panel (Life Technologies). Sequencing was performed on the Ion Proton sequencer (Life Technologies) according to the manufacturer’s protocol. Raw reads were mapped to the targeted fusion transcripts using BWA (Burrows-Wheeler Aligner) software and using the in-house script to identify reads covered the fusion breakpoint.

### Analysis of TCGA public data

Cases from TCGA were selected from the lung adenocarcinoma (TCGA, Provisional). A total of 313 stage I/II patients were selected (Supplementary Table 4). Alteration types of single nucleotide variant, heterozygous and homozygous deletions were all included in a mutant group.

### Statistical analysis

RFS was defined from the date of first surgery until tumor progression, death, the end of follow-up or the end of the study. Survival analysis was conducted to correlate genomic alterations with time to NSCLC relapse using the Kaplan–Meier curve and Gehan-Breslow-Wilcoxon test (v.6.0; GraphPad Inc.). Multivariate Cox regression modeling was performed using potential risk factors (age, gender, tumor stage, smoking and mutation status of genes of interest) by SPSS Version 23.0 (SPSS Inc., Chicago, IL, USA). All calculations were two-sided tests, with a *p* value < 0.05 considered as statistically significant.

## SUPPLEMENTARY MATERIALS TABLES







## Abbreviations

CNA: Copy number alteration
CNV: Copy number variant
ER: Early relapse
FFPE: Formalin-fixed paraffin-embedded
HH: Hedgehog
LOF: Loss-of-function
LR: Late relapse
Mb: Megabase
NGS: Next-generation sequencing
NR: No relapse
NSCLC: Non-small cell lung cancer
PBMC: Peripheral blood mononuclear cell
RFS: Relapse-free survival
TCGA: The Cancer Genome Atlas
TMB: Tumor mutational burden
VUS: Variant of unknown significance
