# Supplementary material for: Targeted next-generation sequencing reveals recurrence-associated genomic alterations in early-stage non-small cell lung cancer
Source: Oncotarget. 2018 Nov 20;9(91):36344–57. doi: 10.18632/oncotarget.26349 (PMC6284742; doi:10.18632/oncotarget.26349)
Supplement: Supplementary file 1 [file oncotarget-09-36344-s001.pdf]

Targeted next-generation sequencing reveals recurrence-associated genomic alterations in early-stage non-small cell lung cancer

SUPPLEMENTARY MATERIALS

Supplementary Table 1: Overview of genomic alterations. See Supplementary_Table_1

Supplementary Table 2: Patient information

Sample ID	Gender	Age	Smoking status	Stage	Relapse	Month & year of first diagnosis	Month & year of relapse
D00807	F	61	Non-smoker	IA (T_1N_0)	Early	Jun-04	Jun-05
D00813	M	72	Ex-smoker	IB ($T_{2a}N_0$)	Early	May-05	Oct-05
D00815	M	66	Ex-smoker	IB ($T_{2a}N_0$)	Early	Dec-04	Jun-05
D00819	M	69	Ex-smoker	IB ($T_{2a}N_0$)	Early	Oct-04	Apr-05
D00823	M	68	Current smoker	IB ($T_{2a}N_0$)	Early	Jul-04	Dec-04
D00825	M	79	Ex-smoker	IB ($T_{2a}N_0$)	Early	Jun-04	Dec-04
D01201	M	72	Ex-smoker	IB ($T_{2a}N_0$)	Early	Oct-14	Apr-15
D01202	F	68	Non-smoker	IB ($T_{2a}N_0$)	Early	Oct-14	Jul-15
D01203	F	75	Non-smoker	IIA ($T_{1a}N_1$)	Early	Feb-14	Feb-15
D01204	M	43	Ex-smoker	IB ($T_{2a}N_0$)	Early	Aug-14	Jul-15
D01205	M	62	Current smoker	IA ($pT_{1a}N_0$)	Early	Dec-14	Sep-15
D01207	F	64	Non-smoker	IIA ($pT_{2a}N_1$)	Early	Apr-14	Feb-15
D01208	F	85	Ex-smoker	IA ($pT_{1a}N_0$)	Early	Apr-14	Aug-14
D01209	F	63	Not mention	IA ($pT_{1b}N_0$)	Early	Mar-15	Feb-16
D01303	M	47	Non-smoker	IB ($pT_{2a}N_0$)	Early	Sep-13	Jan-14
D01304	M	57	Ex-smoker	IB ($T_{2a}N_0$)	Early	Aug-13	May-14
D01004	M	77	Non-smoker	IB ($T_{2a}N_0$)	Late	Jan-04	Sep-13
D01006	M	74	Non-smoker	IIB (T_2N_1)	Late	Mar-04	May-05
D01008	F	57	Non-smoker	IB ($T_{2a}N_0$)	Late	Mar-04	Nov-11
D01010	F	68	Non-smoker	IB ($T_{2a}N_0$)	Late	Apr-04	Nov-13
D01014	M	76	Ex-smoker	IB ($T_{2a}N_0$)	Late	May-04	Jan-15
D01016	M	52	Non-smoker	IIB (T_2N_1)	Late	Nov-04	Jun-06
D01018	F	47	Non-smoker	IA (T_1N_0)	Late	Oct-05	Jan-12
D01192	F	70	Non-smoker	IA (T_1N_0)	Late	Jan-14	Jun-15
D01197	M	63	Ex-smoker	IA ($T_{1b}N_0$)	Late	Oct-14	Apr-16
D01199	M	54	Current smoker	IA ($pT_{1b}N_0$)	Late	Jun-14	Jun-17
D01206	F	62	Non-smoker	IA ($pT_{1b}N_0$)	Late	Sep-14	Oct-15
D01302	M	65	Ex-smoker	IA ($pT_{1b}N_0$)	Late	Sep-13	Jan-15
D01305	M	71	Ex-smoker	IB ($pT_{2a}N_0$)	Late	Dec-13	Sep-16
D01306	F	61	Non-smoker	1B (pT_2N_0)	Late	Nov-13	Nov-16
D01307	F	71	Non-smoker	IA ($pT1_bN_0$)	Late	Oct-13	Dec-15
D00803	M	62	Current smoker	IA (T_1N_0)	No	Jun-04	Not applicable
D00805	F	64	Ex-smoker	IA (T_1N_0)	No	May-05	Not applicable
D00811	M	58	Non-smoker	IB ($T_{2a}N_0$)	No	Mar-04	Not applicable
D00821	M	73	Ex-smoker	IB ($T_{2a}N_0$)	No	Oct-04	Not applicable
D01191	F	81	Non-smoker	IA (T_1N_0)	No	Oct-14	Not applicable
D01193	M	81	Ex-smoker	IA (pT_{1b})	No	Sep-14	Not applicable
D01194	M	65	Current smoker	IA (T_1N_0)	No	Feb-14	Not applicable
D01195	M	65	Non-smoker	IA (T_1N_0)	No	Oct-14	Not applicable
D01196	F	76	Non-smoker	IA ($T_{1b}N_0$)	No	Jul-14	Not applicable
D01198	M	53	Ex-smoker	1A (T_1N_0)	No	Jun-14	Not applicable
D01200	M	59	Ex-smoker	1A ($pT_{1a}N_0$)	No	Feb-14	Not applicable

Supplementary Table 3: Complete list of all 440 genes included in the study

ABCB1	AURKB	CBL	CDKN2B	E2F3	FAT1	GRIN2A	JAK2	MED12	NOTCH4	PMS1	RAD51D	SLCO1B3	TNFRSF14
ABCC2	AXIN1	CCNA1	CDKN2C	EGFR	FBXW7	GSK3B	JAK3	MEF2B	NPM1	PMS2	RAD52	SMAD2	TNFSF11
ABCG2	AXIN2	CCNA2	CEBPA	EP300	FCGR2B	GSTP1	JUN	MEN1	NQO1	POLB	RAD54L	SMAD3	TOP1
ABL1	AXL	CCNB1	CHEK1	EPCAM	FGF1	GSTT1	KAT6A	MET	NRAS	POLD1	RAF1	SMAD4	TP53
ABL2	B2M	CCNB2	CHEK2	EPHA2	FGF10	HGF	KDM5A	MITF	NSD1	POLE	RARA	SMARCA4	TPMT
ADAMTS1	BAP1	CCNB3	CIC	EPHA3	FGF14	HIF1A	KDM5C	MLH1	NTRK1	PPARG	RB1	SMARCB1	TSC1
ADAMTS13	BARD1	CCND1	CREBBP	EPHA5	FGF19	HIST1H1C	KDM6A	MPL	NTRK2	PPP2R1A	RBM10	SMO	TSC2
ADAMTS15	BCL10	CCND2	CRKL	EPHA7	FGF23	HIST1H1E	KDR	MRE11	NTRK3	PRDM1	RECQL4	SOCS1	TSHR
ADAMTS16	BCL2	CCND3	CRLF2	EPHB1	FGF3	HNF1A	KEAP1	MSH2	PAK3	PRKAR1A	REL	SOX2	TYMS
ADAMTS18	BCL2L1	CCNE1	CSF1R	ERBB2	FGF4	HR	KIT	MSH6	PALB2	PRKCA	RET	SOX9	U2AF1
ADAMTS6	BCL2L2	CCNE2	CTCF	ERBB3	FGF6	HRAS	KMT2A	MTHFR	PARP1	PRKCB	RHOA	SPEN	UBE2A
ADAMTS9	BCL6	CCNH	CTLA4	ERBB4	FGFR1	HSP90AA1	KMT2C	MTOR	PAX5	PRKCG	RICTOR	SPOP	UBE2K
ADAMTSL1	BCL9	CD19	CTNNA1	ERCC1	FGFR2	HSP90AB1	KMT2D	MUC16	PAX8	PRKCI	RNF43	SRC	UBR5
ADGRA2	BCOR	CD274	CTNNB1	ERCC2	FGFR3	HSPA4	KRAS	MUC4	PBRM1	PRKCQ	ROS1	STAG2	UGT1A1
ADH1C	BIRC2	CD58	CUL3	ERCC3	FGFR4	HSPA5	LCK	MUC6	PDCD1	PRKDC	RPPH1	STAT3	USH2A
AKT1	BIRC3	CD70	CYLD	ERCC4	FH	IDH1	LIG1	MUTYH	PDCD1LG2	PRKN	RPTOR	STK11	VDR
AKT2	BLM	CD79A	CYP1A1	ERCC5	FLCN	IDH2	LIG3	MYC	PDGFRA	PSMB8	RUNX1	SUFU	VEGFA
AKT3	BMPR1A	CD79B	CYP2B6	ERG	FLT1	IFNL3	LMO1	MYCL	PDGFRB	PSMB9	RUNX1T1	SYK	VEGFB
ALDH1A1	BRAF	CDC73	CYP2C19	ESR1	FLT3	IGF1	LRP1B	MYCN	PDIA3	PSME1	RXRA	SYNE1	VHL
ALK	BRCA1	CDH1	CYP2C8	ESR2	FLT4	IGF1R	LYN	MYD88	PGF	PSME2	SDHA	TAF1	WT1
AMER1	BRCA2	CDK1	CYP2D6	ETV1	FOXL2	IGF2	MALT1	NAT2	PHOX2B	PSME3	SDHB	TAP1	XIAP
APC	BRD4	CDK12	CYP2E1	ETV4	FOXP1	IKBKB	MAP2K1	NBN	PIK3C2B	PTCH1	SDHC	TAP2	XPO1
AR	BRIP1	CDK2	CYP3A4	EZH2	FRG1	IKBKE	MAP2K2	NEFH	PIK3C2G	PTEN	SDHD	TAPBP	XRCC2
ARAF	BTG1	CDK4	CYP3A5	FAM46C	FUBP1	IKZF1	MAP2K4	NF1	PIK3C3	PTGS2	SERPINB3	TBX3	ZNF217
ARID1A	BTG2	CDK5	DAXX	FANCA	GATA1	IL6	MAP3K1	NF2	PIK3CA	PTPN11	SERPINB4	TEK	
ARID1B	BTK	CDK6	DCUN1D1	FANCC	GATA2	IL7R	MAP3K7	NFE2L2	PIK3CB	PTPRD	SETD2	TERT	
ARID2	BUB1B	CDK7	DDR2	FANCD2	GATA3	INPP4B	MAPK1	NFKB1	PIK3CD	PTPRT	SF3B1	TET1	
ASXL1	CALR	CDK8	DICER1	FANCE	GNA11	INSR	MAPK3	NFKBIA	PIK3CG	RAC1	SGK1	TET2	
ATM	CANX	CDK9	DNMT3A	FANCF	GNA13	IRF4	MAX	NKX2-1	PIK3R1	RAD50	SH2D1A	TGFBR2	
ATR	CARD11	CDKN1A	DOT1L	FANCG	GNAQ	IRS1	MCL1	NOTCH1	PIK3R2	RAD51	SLC19A1	TMSB4X	
ATRX	CASP8	CDKN1B	DPYD	FANCL	GNAS	IRS2	MDM2	NOTCH2	PIK3R3	RAD51B	SLC22A2	TNF	
AURKA	CBFB	CDKN2A	DTX1	FAS	GREM1	JAK1	MDM4	NOTCH3	PIM1	RAD51C	SLCO1B1	TNFAIP3	

Supplementary Table 4: Seleted patients from TCGA data (TCGA, provisional). See Supplementary_Table_4
